# Supplementary material for: Muscle 4EBP1 activation modifies the structure and function of the neuromuscular junction in mice
Source: Nat Commun. 2022 Dec 17;13:7792. doi: 10.1038/s41467-022-35547-0 (PMC9758177; doi:10.1038/s41467-022-35547-0)
Supplement: Supplementary file 1 — Supplementary Information [file 41467_2022_35547_MOESM1_ESM.pdf]

Supplemental information

Title: Muscle 4EBP1 activation modifies the structure and function of the neuromuscular junction in mice

Authors and affiliations:

Seok-Ting J. Ang[1,2], Elisa M. Crombie[1], Han Dong[1], Kuan-Ting Tan[1], Adriel Hernando[1], Dejie Yu[2,3,4], Stuart Adamson[5], Seonyoung Kim[1], Dominic J. Withers[6,7], Hua Huang[2,3,4] and Shih-Yin Tsai[1,2]

[1]Department of Physiology, Yong Loo Lin School of Medicine, National University of Singapore, Singapore, 117593, Singapore.

[2]Healthy Longevity Translational Research Programme, Yong Loo Lin School of Medicine, National University of Singapore, Singapore, 117456, Singapore

[3]Electrophysiology Core Facility, Yong Loo Lin School of Medicine, National University of Singapore, Singapore 117544, Singapore

[4]Cardiovascular Diseases Program, National University of Singapore, Singapore 117599, Singapore

[5]Buck Institute for Research on Aging, Novato, California, USA

[6]Metabolic Signalling Group, Medical Research Council Clinical Council London Institute of Medical Sciences (LMS), Du Cane Road, London W12 0NN, United Kingdom.

[7]Institute of Clinical Sciences (ICS), Faculty of Medicine, Imperial College London, Du Cane Road, London W12 0NN, United Kingdom.

This PDF file includes:

Supplementary Figure 1-7

Supplementary Table 1-7

Supplementary Figures

Supplementary Fig. 1: Molecular and histological analysis of the NMJ.

a. Quantification of the percentage of denervated NMJs in 6- and 28-month-old control male mice (using values from Fig. 1d). A denervated NMJ is defined as Mander's colocalization coefficient lower than 0.4. Sample size: 6mo Control, n=8 and 28mo Control, n=10; on average, 25 NMJs were analyzed per mouse. Statistical significance was determined by two-tailed unpaired student t-test.

b. qPCR of select AChR subunit and denervation-related genes in whole muscle lysate from 6- and 28- month-old control mice. Sample size: 6mo Control Female, n=3-5; 28mo Control Female, n=5-6; 6mo Control Male, n=3-4; 28mo Control Male, n=6-7. Statistical significance was determined by one-way ANOVA followed by Tukey's post-hoc pairwise comparison. Only a P value of less than 0.05 would be labeled in the figure.

c. Immunoblotting in quadriceps muscle of 12-month-old control and TSC1mKO mice. Ponceau red staining accompanying the immuoblots showing equal protein loading. Each lane represents individual mouse sample. Sample size: Control Male, n=4; TSC1mKO Male, n=4; Control Female, n=3; TSC1mKO Female, n=3.

d. Percentage distribution of AChR fragments of 12-month-old control (abbreviated as **C**), 12-month-old TSC1mKO (abbreviated as **T**), and 28-month-old control (abbreviated as **28C**) mice (analysis from Fig. 3c). Sample size: 12mo Control Female, n=3; 12mo TSC1mKO Female, n=4; 28mo Control Female, n=7; 12mo Control Male, n=4; 12mo TSC1mKO Male, n=3; 28mo Control Male, n=10; on average, 25 NMJs were analyzed per mouse. Statistical significance was determined by two-way ANOVA followed by Tukey multiple comparisons test. Only a P value of less than 0.05 would be labeled in the figure.

e. qPCR of select AChR subunit and denervation-related genes in whole muscle lysate from 12-month-old control and TSC1mKO mice. Sample size: 12mo Control Female, n=4-5; 12mo TSC1mKO Female, n=5-6; 12mo Control, n=5-7; TSC1mKO Male, n=5. Statistical significance was

determined by one-way ANOVA followed by Tukey's post-hoc pairwise comparison. Only a P value of less than 0.05 would be labeled in the figure.

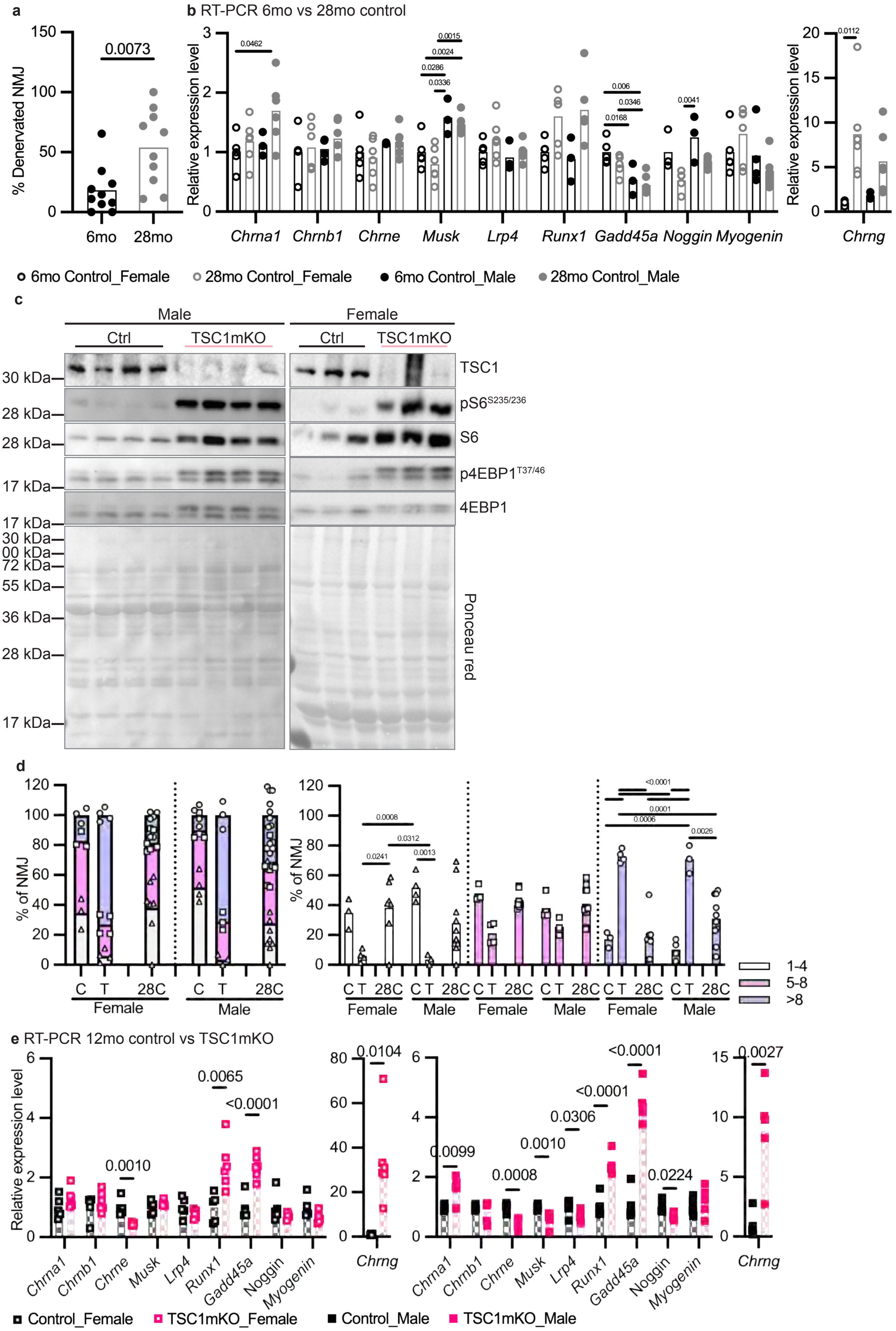
a
% Denervated NMJ
0.0073
6mo
28mo
b RT-PCR 6mo vs 28mo control
Relative expression level
Chrna1
Chrnb1
Chrne
Musk
Lrp4
Runx1
Gadd45a
Noggin
Myogenin
Chrng
6mo Control_Female
28mo Control_Female
6mo Control_Male
28mo Control_Male
c
Male
Female
Ctrl
TSC1mKO
TSC1
pS6S235/236
S6
p4EBP1T37/46
4EBP1
Ponceau red
d
% of NMJ
Female
Male
1-4
5-8
>8
e RT-PCR 12mo control vs TSC1mKO
Relative expression level
Control_Female
TSC1mKO_Female
Control_Male
TSC1mKO_Male

Figure S1

Supplementary Fig. 2: Myofiber analysis of S6K1mKO mice.

a-b. Representative H&E staining of (**a**) soleus and (**b**) tibialis anterior muscle from 6-month-old male mice (Control, n=4; S6K1mKO, n=3). Scale bars are indicated in the image.

c-d. Immunofluorescent staining for MyHC and WGA (white) (**c**) soleus (scale bar = 100μm) and (**d**) tibialis anterior (scale bar = 200μm) muscle from 6-month-old male mice (Control, n=4; S6K1mKO, n=3).

e-f. Quantification of cross-section size from selective MyHC in (**e**) soleus and (**f**) tibialis anterior muscle from male mice. Sample size: 6mo Control, n=3-4; 6mo S6K1mKO, n=3; 28mo Control, n=5; 28mo S6K1mKO, n=3-4; on average, ~6000-11000 myofibers were analyzed per mouse. Statistical significance: Data are shown as mean ± SEM and statistical significance was determined by two-way ANOVA with Tukey's multiple comparison test compared within the size class. *P<0.05, **P<0.01, ***P<0.001, ****P<0.0001 indicates statistical significance of young versus old in either Control (black) or S6K1mKO (light blue) mice. #P<0.05, ##P<0.01, ###P<0.001, indicates significance of Control versus S6K1mKO in young (gray) mice.

g. Measurement of muscle coordination and strength by four limbs hanging test in 4-month-old male and female mice. Sample size: Control Male, n=20; S6K1mKO Male, n=26; Control Female, n=16; S6K1mKO Female, n=20. Statistical significance was determined by one-way ANOVA with Tukey's multiple comparison test compared within the same sex group. Only P<0.05 will be indicated in the graph.

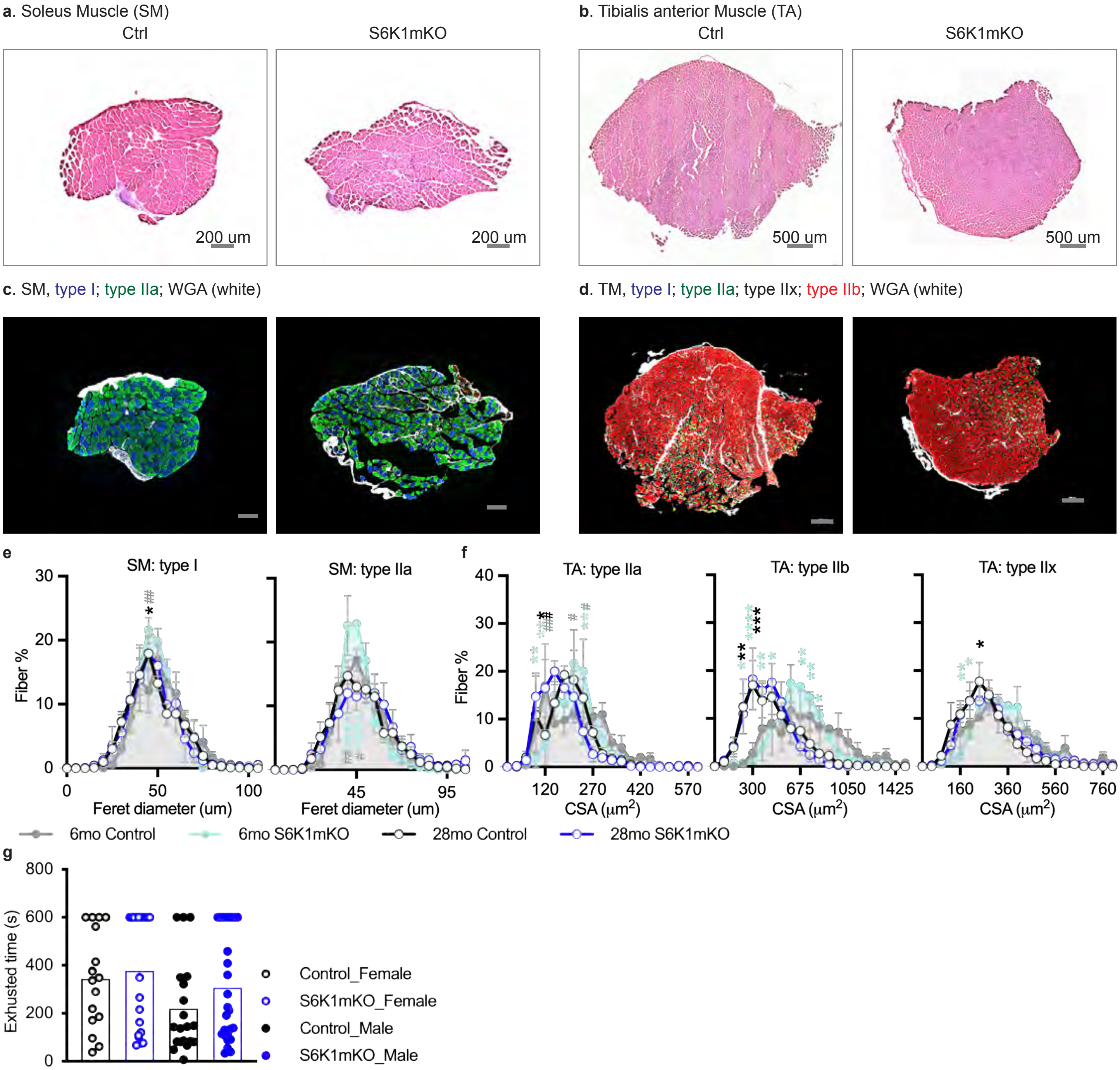
a. Soleus Muscle (SM)
Ctrl
S6K1mKO
200 um
200 um
b. Tibialis anterior Muscle (TA)
Ctrl
S6K1mKO
500 um
500 um
c. SM, type I; type IIa; WGA (white)
d. TM, type I; type IIa; type IIx; type IIb; WGA (white)
e
SM: type I
SM: type IIa
Fiber %
Feret diameter (um)
Feret diameter (um)
f
TA: type IIa
TA: type IIb
TA: type IIx
CSA (μm2)
6mo Control
6mo S6K1mKO
28mo Control
28mo S6K1mKO
g
Exhusted time (s)
Control_Female
S6K1mKO_Female
Control_Male
S6K1mKO_Male

Figure S2

Supplementary Fig. 3: Muscle-specific deletion of *S6k1* attenuates intracellular pS6 accumulation during aging.

a. Representative confocal images of BTX (green), $pS6^{S240+244}$ (red), DAPI (blue) and Laminin (white) staining from cross-sections of the gastrocnemius muscle of 6-month-old control (n=3) and S6K1mKO (n=3) male mice. The BTX and $pS6^{S240+244}$ channels are shown in the bottom panels. Open arrow = junctional $pS6^{S240+244}$; Arrowhead = $pS6^{S240+244}$ in the nucleus. Scale bar = 50µm.

b. Representative confocal images of BTX (green), $pS6^{S240+244}$ (red), DAPI (blue) and Laminin (white) staining from cross-sections of the gastrocnemius muscle of 12- and 28-month-old male mice (12mo S6K1-TSC1mKO, n=3; 28mo S6K1mKO, n=5). The insets are shown in the bottom panels. Asterisks = intracellular $pS6^{S240+244}$ in angular, odd-shaped fibers; Arrowhead = $pS6^{S240+244}$ in the nucleus, including central nucleus. Scale bars = 50µm.

c. Quantification of the percentage of $pS6^{S240+244}$-positive myofibers (left panel, one-way ANOVA followed by Tukey's post-hoc pairwise comparison) and mean CSA of $pS6^{S240+244}$ -positive (pS6+) and $pS6^{S240+244}$ -negative (pS6-) fibers (right panel, two-way ANOVA followed by Tukey's post-hoc pairwise comparison**)** in the gastrocnemius muscle sampled from 6-, 12-, and 28-month-old male mice. Sample size: 6mo Control and S6K1mKO Male, n=3 per group; 28mo Control Male, n=6; 28mo S6K1mKO Male, n=5; 12mo Control, TSC1mKO, and S6K1-TSC1mKO Male, n=3 per group; on average, 800 myofibers were analyzed per mouse. Detailed statistical analysis of CSA between groups within pS6- and pS6+ fibers is listed in **Supplementary Table. 1**.

d. Immunoblotting of S6 phosphorylation at Ser240+244 and total S6 as well as total S6K1 in gastrocnemius muscle of 4-month-old control, S6K1mKO, TSC1mKO, and S6K1-TSC1mKO male mice. Ponceau red staining accompanying the immuoblots showing equal protein loading. Each lane represents individual mouse sample. Sample size: Control, n=11; S6K1mKO, n=4; TSC1mKO, n=4; TSC1-S6K1mKO, n=3; S6K1cko, n=1.

e. Immunoblotting of S6K2 phosphorylation at Ser423 and total S6K2 in gastrocnemius muscle of 4-month-old control and S6K1mKO male mice. Ponceau red staining accompanying the immuoblots showing equal protein loading. Each lane represents individual mouse sample. Sample size: Control, n=4; S6K1mKO, n=4; S6K1cko, n=1.

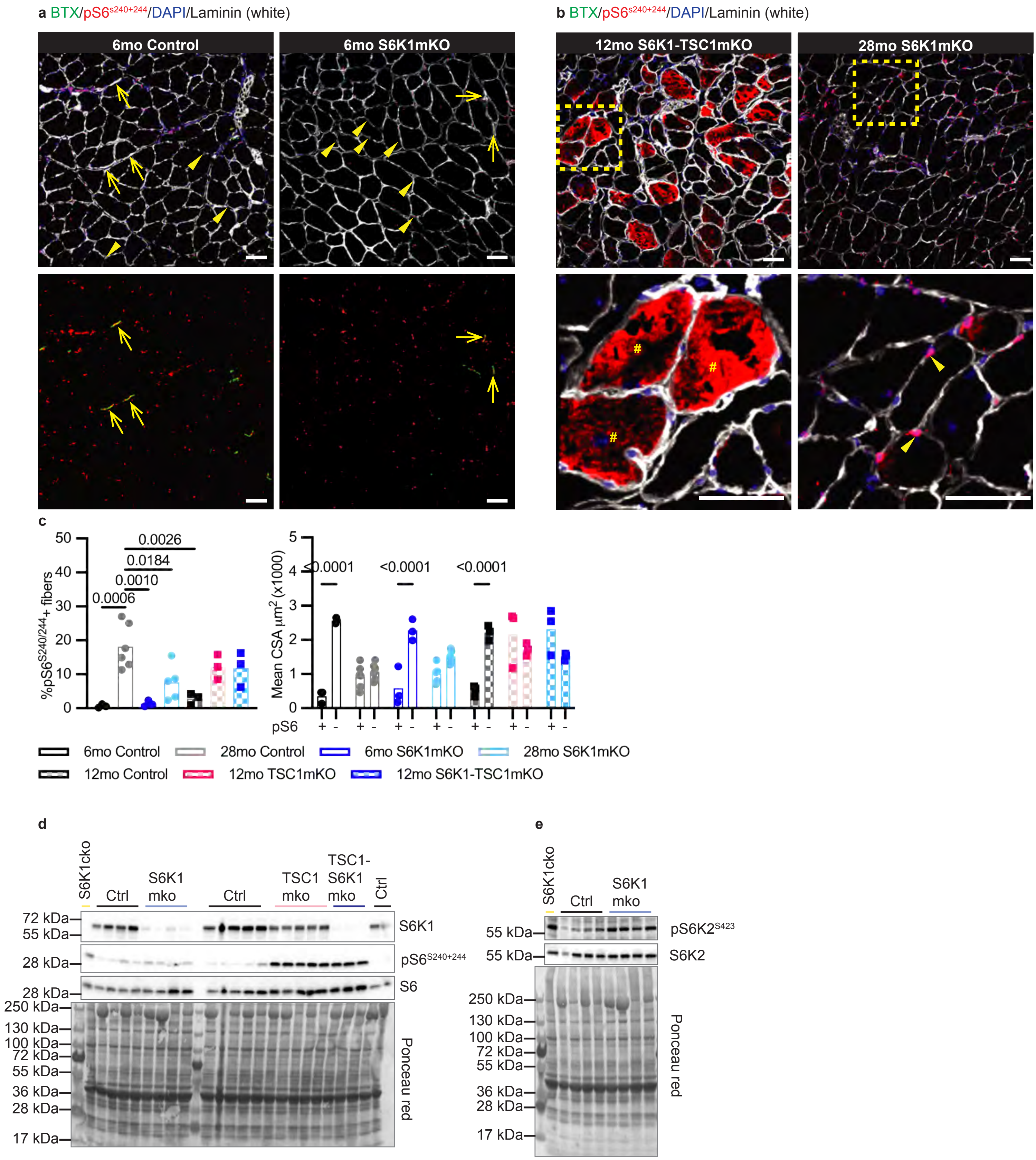
a BTX/pS6s240+244/DAPI/Laminin (white)
6mo Control
6mo S6K1mKO
b BTX/pS6s240+244/DAPI/Laminin (white)
12mo S6K1-TSC1mKO
28mo S6K1mKO
c
%pS6S240/244+ fibers
0.0026
0.0184
0.0010
0.0006
Mean CSA µm2 (x1000)
<0.0001
<0.0001
<0.0001
pS6
6mo Control
28mo Control
6mo S6K1mKO
28mo S6K1mKO
12mo Control
12mo TSC1mKO
12mo S6K1-TSC1mKO
d
S6K1cko
Ctrl
S6K1 mko
Ctrl
TSC1 mko
TSC1-S6K1 mko
Ctrl
S6K1
pS6S240+244
S6
Ponceau red
e
S6K1cko
Ctrl
S6K1 mko
pS6K2S423
S6K2
Ponceau red

Figure S3

Supplementary Fig. 4: Muscle-specific deletion of *S6k1* has no effect on NMJ remodeling.

a. Representative confocal images of NMJs from longitudinal sections of the quadriceps muscle of male mice (28mo Control, n=10; 28mo S6K1mKO, n=4; 12mo Control, n=4; 12mo TSC1mKO and S6K1-TSC1mKO, n=3 per group). Top panels = BTX (green) and neurofilament (NF; red) + synaptophysin (Syn; red); Bottom panels = BTX (green) and DAPI (blue). Scale bar = 20µm.

b-e. Quantification of NMJ morphological properties which include **(b)** the number of AChR fragmentation, **(c)** the number of post-synaptic myonuclei, (**d**) Mander's colocalization coefficient, and (**e**) quantification of the percentages of NCAM-positive myofibers.

f. Representative confocal images of BTX (green), NCAM (red), DAPI (blue) and Laminin (white) staining from cross-sections of the gastrocnemius muscle of male mice (6mo Control, n=3; 28mo Control, n=6; 28mo S6K1mKO, n=5; 12mo Control, TSC1mKO, and S6K1-TSC1mKO Male, n=3 per group). Scale bars = 50µm.

Sample size: NMJ quantification (Supplementary Fig. 4b-d), 6mo and 28mo Control Female, n=7 per group; 6mo and 28mo S6K1mKO Female, n=3 per group; 12mo Control and S6K1-TSC1mKO Female, n=3 per group; 12mo TSC1mKO Female, n=4; 6mo and 28mo Control Male, n=10 per group; 6mo and 28mo S6K1mKO Male, n=4 per group; 12mo Control Male, n=4; 12mo TSC1mKO and S6K1-TSC1mKO Male, n=3 per group; on average, 25 NMJs were analyzed per animal. Myofiber analysis of NCAM (Supplementary Fig. 4e), 6mo Control and S6K1mKO Male, n=3 per group; 28mo Control Male, n=6; 28mo S6K1mKO Male, n=5; 12mo Control, TSC1mKO, and S6K1-TSC1mKO Male, n=3 per group; on average, 800 myofibers were analyzed per mouse.

Statistical significance: NMJ quantification (Supplementary Fig. 4b-d), two-way ANOVA followed by Tukey's post-hoc pairwise comparison; Myofiber analysis of NCAM (Supplementary Fig. 4e), one-way ANOVA followed by Tukey's post-hoc pairwise comparison. Only $P<0.05$ will be indicated in the graph.

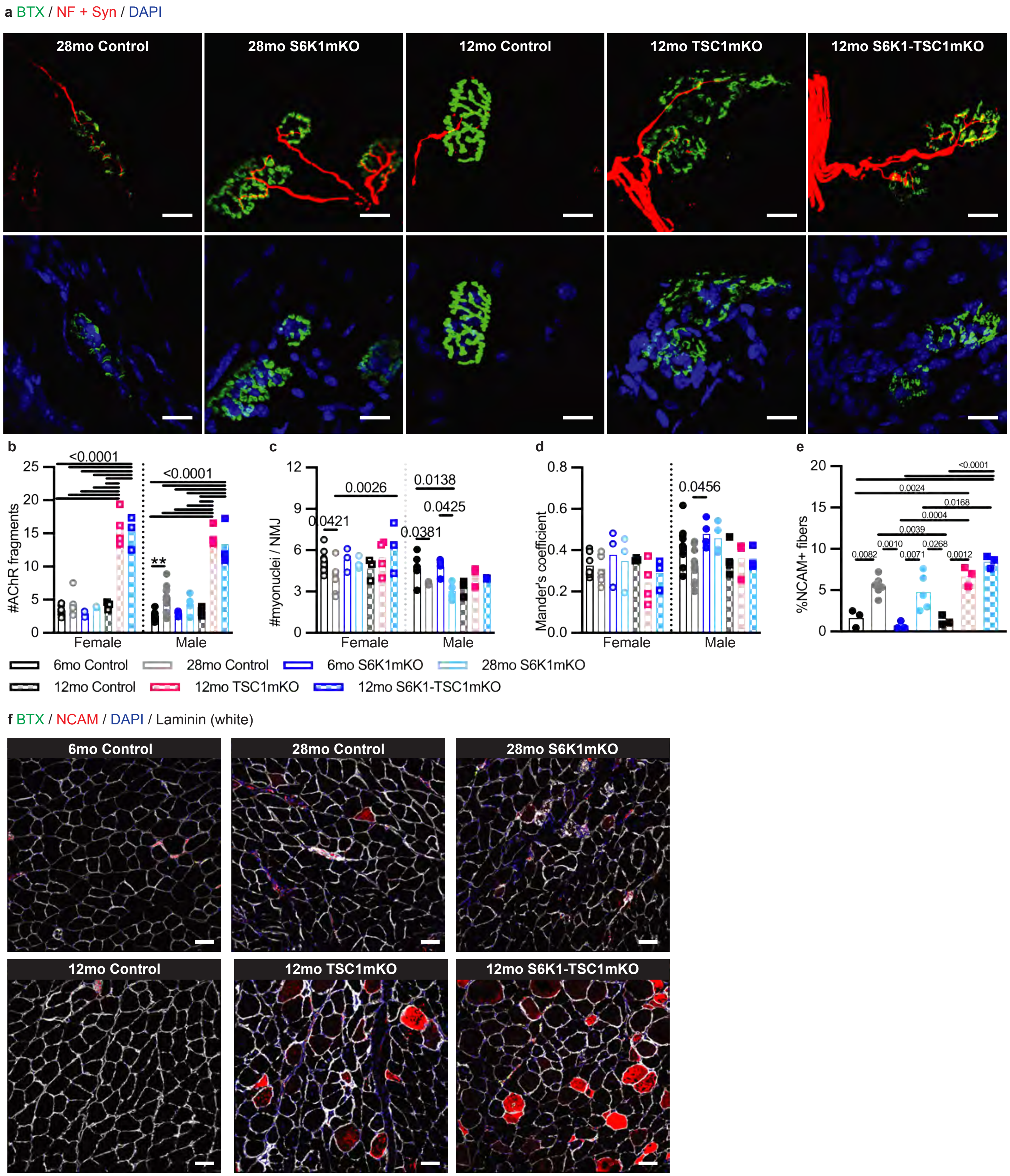
a BTX / NF + Syn / DAPI
28mo Control
28mo S6K1mKO
12mo Control
12mo TSC1mKO
12mo S6K1-TSC1mKO
b
#AChR fragments
<0.0001
<0.0001
**
Female
Male
c
#myonuclei / NMJ
0.0026
0.0421
0.0138
0.0425
0.0381
Female
Male
d
Mander's coefficient
0.0456
Female
Male
e
%NCAM+ fibers
<0.0001
0.0024
0.0168
0.0004
0.0039
0.0082
0.0010
0.0071
0.0268
0.0012
6mo Control
28mo Control
6mo S6K1mKO
28mo S6K1mKO
12mo Control
12mo TSC1mKO
12mo S6K1-TSC1mKO
f BTX / NCAM / DAPI / Laminin (white)
6mo Control
28mo Control
28mo S6K1mKO
12mo Control
12mo TSC1mKO
12mo S6K1-TSC1mKO

Figure S4

Supplementary Fig. 5: Molecular and histological analysis of NMJ with activation of 4EBP1 in the skeletal muscle.

a-b. Quantification of (**a**) endplate area and (**b**) AChR area of 6-month-old male control (n=5) and 4EBP1mt-muscle (n=3).

c-d. Percentage distribution of (**c**) number of fragments from further analysis of Fig. 4d, and (**d**) number of post-synaptic myonuclei from further analysis of Fig. 4e of 6-month-old and 28-month-old male control and 4EBP1mt-muscle, as well as 12-month-old male control (abbreviated as **C**), TSC1mKO (abbreviated as **T**) and 4EBP1mt-TSC1mKO (abbreviated as **ET**). Sample size: 6mo and 28mo Control , n=10 per group; 6mo 4EBP1mt-muscle, n=4; 28mo 4EBP1mt-muscle, n=5; 12mo Control and 4EBP1mt-TSC1mKO, n=4 per group; 12mo TSC1mKO, n=3 per group; on average, 25 NMJs were analyzed per animal.

e-g. qPCR of select AChR subunit genes and denervation-related genes in whole muscle lysate from (**e**) 6-month-old male control (n=4) and 4EBP1mt-muscle (n=3); (**f**) 12-month-old female control (n=4-5), TSC1mKO (n=5-6), S6K1-TSC1mKO (n=6-7), and 4EBP1mt-TSC1mKO (n=4); (**g**) 12-month-old male control (n=5-7), TSC1mKO (n=5), S6K1-TSC1mKO (n=4), and 4EBP1mt-TSC1mKO (n=4).

h. Immunoblotting in gastrocnemius muscle of 12-month-old female control, TSC1mKO, 4EBP1mt-TSC1mKO, and S6K1-TSC1mKO (n=3 per group). Ponceau red staining accompanying the immuoblots showing equal protein loading. Each lane represents individual mouse sample.

Statistical significance: Two-tailed unpaired student t-test for Supplementary Fig. 5a, 5b, and 5e. One-way ANOVA followed by Tukey post-hoc pairwise comparison for Supplementary Fig. 5f and 5g. Only $P<0.05$ will be indicated in the graph.

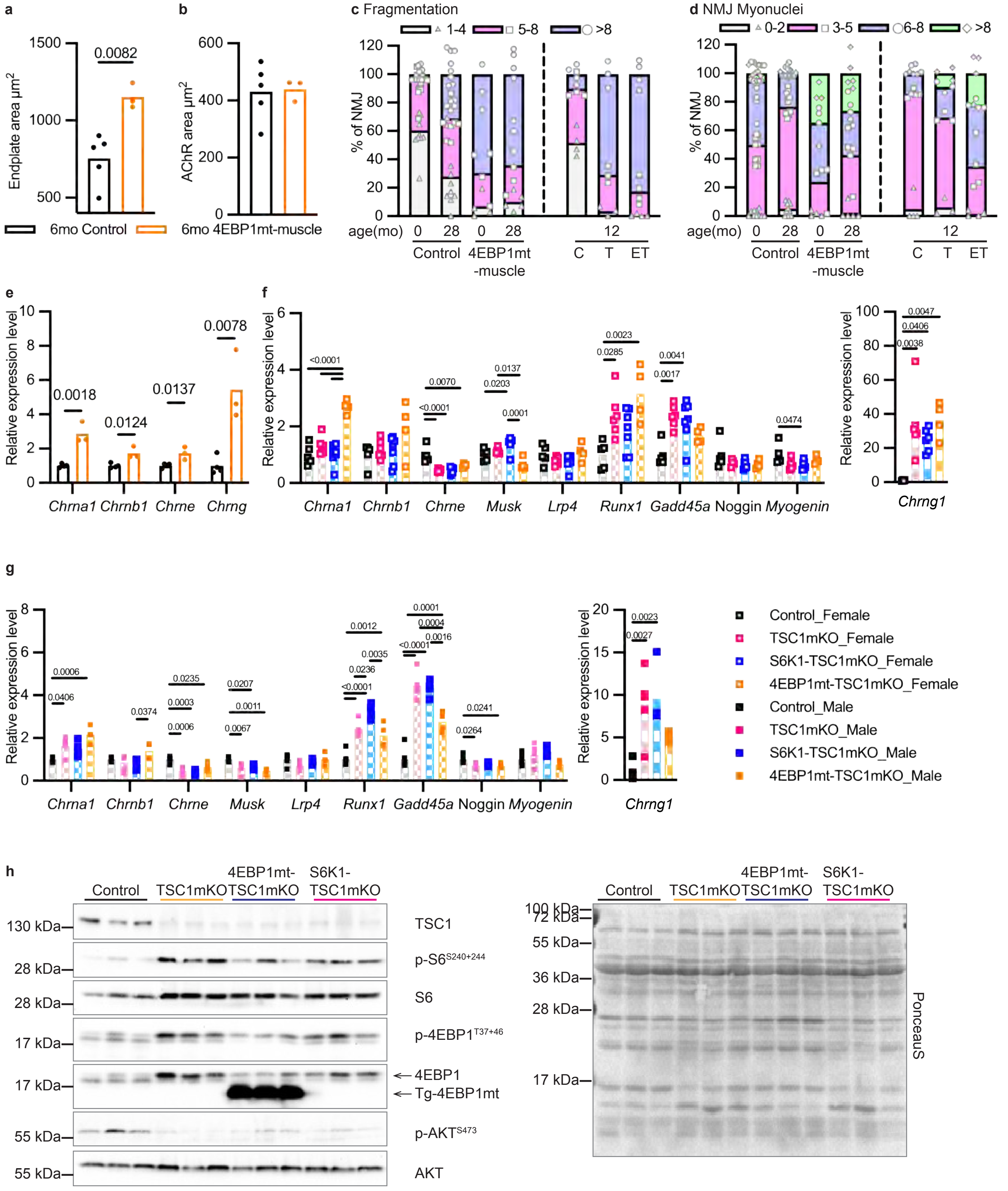
a
b
c Fragmentation
d NMJ Myonuclei
Endplate area μm²
AChR area μm²
6mo Control
6mo 4EBP1mt-muscle
% of NMJ
age(mo)
Control
4EBP1mt -muscle
e
f
g
h
Relative expression level
Chrna1 Chrnb1 Chrne Chrng
Chrna1 Chrnb1 Chrne Musk Lrp4 Runx1 Gadd45a Noggin Myogenin
Chrng1
Control_Female
TSC1mKO_Female
S6K1-TSC1mKO_Female
4EBP1mt-TSC1mKO_Female
Control_Male
TSC1mKO_Male
S6K1-TSC1mKO_Male
4EBP1mt-TSC1mKO_Male
Control
TSC1mKO
4EBP1mt-TSC1mKO
S6K1-TSC1mKO
TSC1
p-S6S240+244
S6
p-4EBP1T37+46
4EBP1
Tg-4EBP1mt
p-AKTS473
AKT
PonceauS

Figure S5

Supplementary Fig. 6: Pathology analysis of TSC1mKO mouse myofiber.

a-b. Representative H&E staining of (**a**) tibialis anterior and (**b**) soleus muscle from 12-month-old male mice (n=4 per group). Black arrows, myofibers with inclusions; white arrows, degenerated basophilic fibers. Scale bar, 50 μm.

c. Measurement of muscle coordination and strength by four limbs hanging test in 4-month-old male and female mice. Sample size: Control Male, n=24; TSC1mKO Male, n=12; TSC1-S6K1mKO Male, n=17; 4EBP1mt-TSC1mKO Male, n=9; Control Female, n=31; TSC1mKO Female, n=16; TSC1-S6K1mKO Female, n=27; 4EBP1mt-TSC1mKO Female, n=6. Statistical significance was determined by one-way ANOVA with Tukey's multiple comparison test of the same sex group. Only $P<0.05$ will be indicated in the graph.

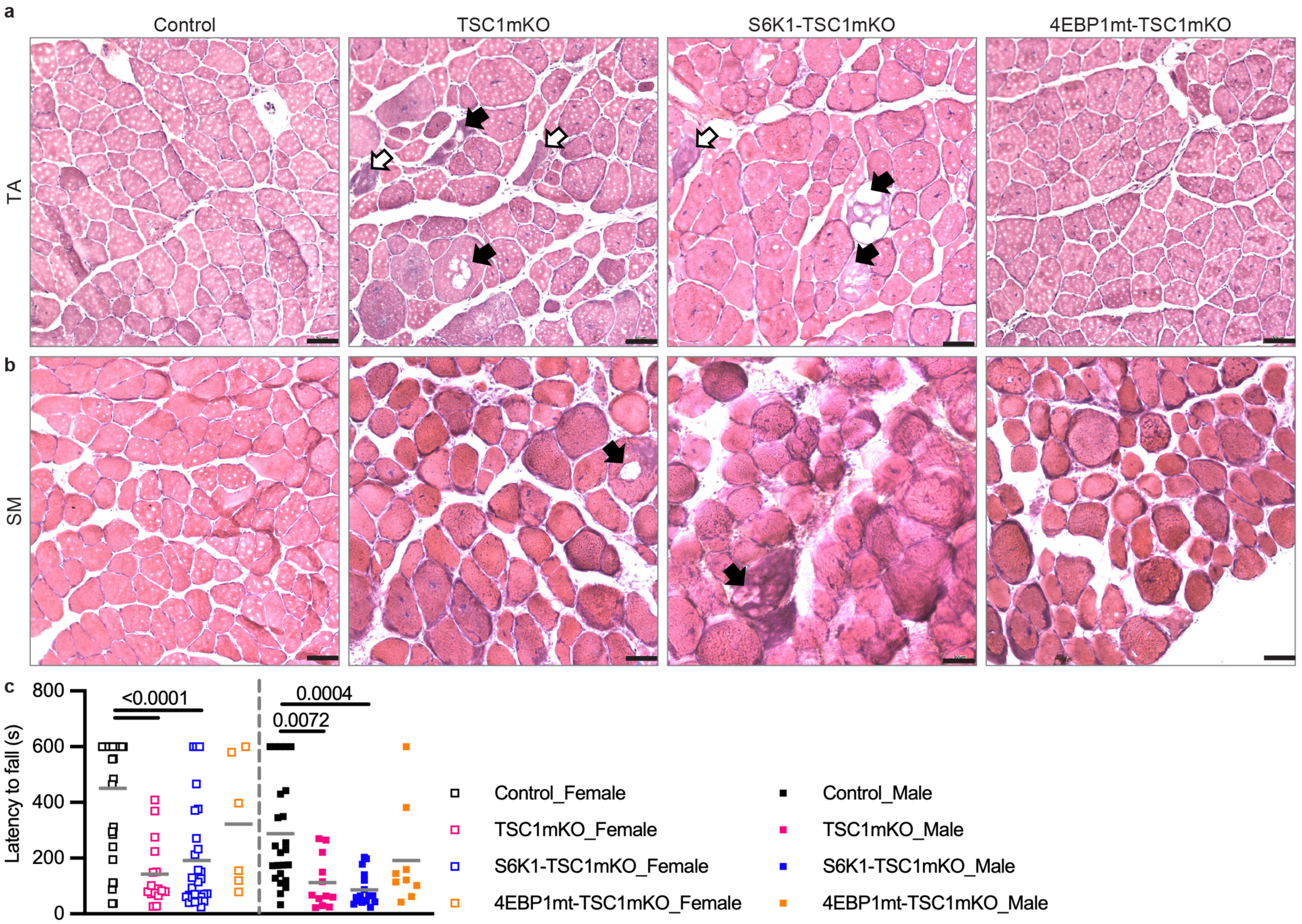

a
Control
TSC1mKO
S6K1-TSC1mKO
4EBP1mt-TSC1mKO
TA
b
SM
c
Latency to fall (s)
800
600
400
200
0
<0.0001
0.0004
0.0072
Control_Female
TSC1mKO_Female
S6K1-TSC1mKO_Female
4EBP1mt-TSC1mKO_Female
Control_Male
TSC1mKO_Male
S6K1-TSC1mKO_Male
4EBP1mt-TSC1mKO_Male

Figure S6

Supplementary Fig. 7: Activation of 4EBP1 in the skeletal muscle enhances NMJ synaptic transmission and myofiber regeneration.

a. Representative confocal images of NMJs from longitudinal sections of the quadriceps muscle from 3-month-old male mice (Control, n=4; 4EBPmt-muscle, n=3). BTX (green) and neurofilament (NF; red) + synaptophysin (Syn; red); Scale bar = 20 μm.

b. Representative traces of miniature endplate potential (mEPP, top) and EPP (bottom) recorded from the diaphragm of 3-month-old male mice (n=6 per group).

c. Normalized EPP (to first pulse) recorded in the diaphragm in response to repeated phrenic nerve stimulation at 10Hz (n=6 per group).

d-e. Percentages of central nucleated fibers sampled from the gastrocnemius muscle of (**d**) 28-month-old control (n=6) and 4EBP1mt-muscle (n=3) male mice, as well as (**e**) 12-month-old control, TSC1mKO, 4EBP1mt-TSC1mKO male mice (n=3 per group). Data are presented as mean values ± SEM. Statistical significance was determined by two-tailed unpaired student t-test for Supplementary Fig. 7d and one-way ANOVA followed by Tukey post-hoc pairwise comparison for Supplementary Fig. 7e. Only P<0.05 will be indicated in the graph.

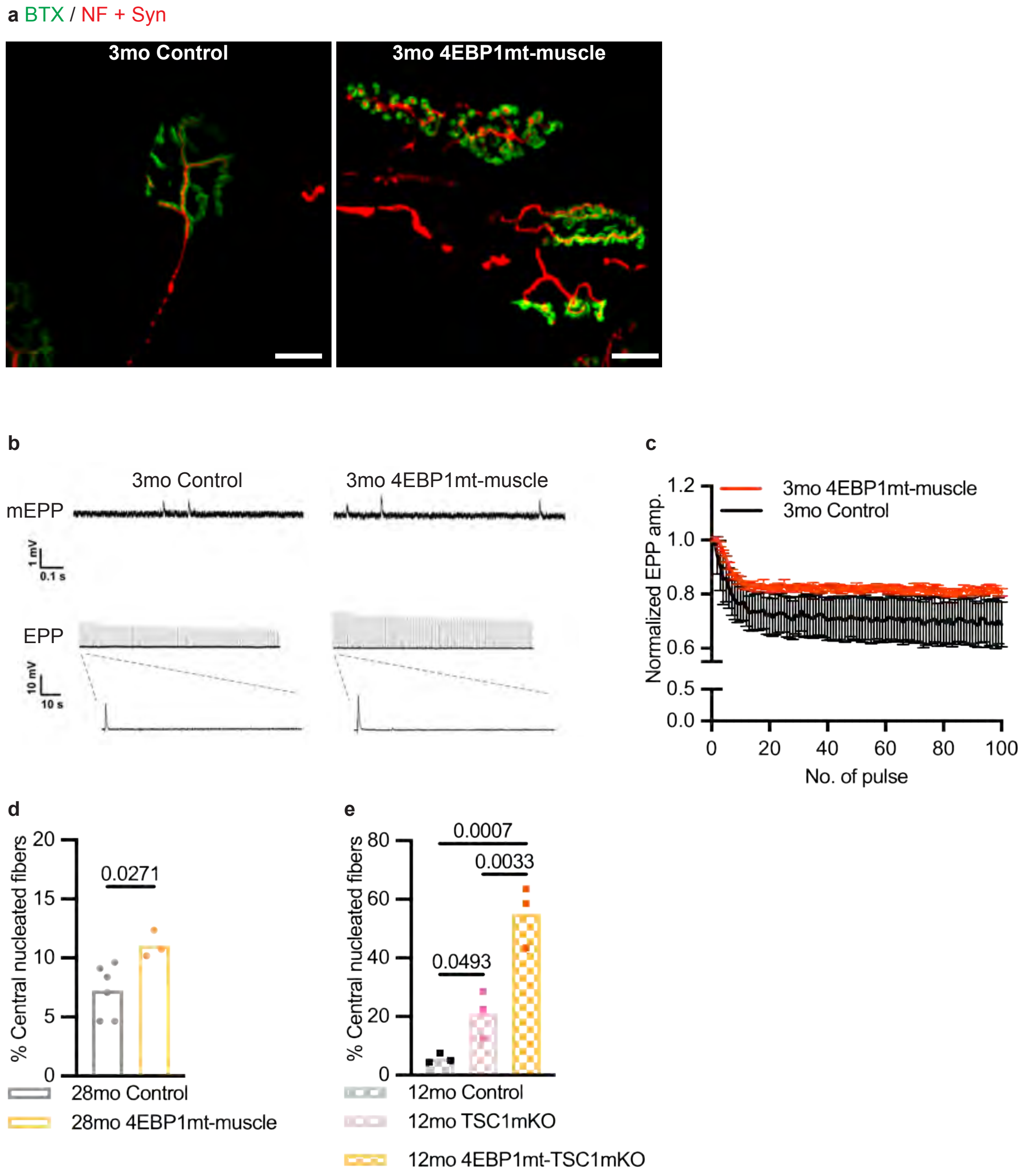
a BTX / NF + Syn
3mo Control
3mo 4EBP1mt-muscle
b
3mo Control
3mo 4EBP1mt-muscle
mEPP
1 mV
0.1 s
EPP
10 mV
10 s
c
3mo 4EBP1mt-muscle
3mo Control
Normalized EPP amp.
No. of pulse
d
% Central nucleated fibers
0.0271
28mo Control
28mo 4EBP1mt-muscle
e
% Central nucleated fibers
0.0007
0.0033
0.0493
12mo Control
12mo TSC1mKO
12mo 4EBP1mt-TSC1mKO

Figure S7

Supplementary Table 1
Statistical analysis of CSA quantification between groups (Supplementary Fig. 3C).

two-way ANOVA pS6+	6mo Control	28mo Control	6mo S6K1mKO	28mo S6K1mKO	12mo Control	12mo TSC1mKO	12mo S6K1-TSC1mKO
**6mo Control**						<0.0001	<0.0001
**28mo Control**						0.004	<0.0001
**6mo S6K1mKO**						<0.0001	<0.0001
**28mo S6K1mKO**						0.014	0.003
**12mo Control**						<0.0001	<0.0001
**12mo TSC1mKO**	<0.0001	0.004	<0.0001	0.014	<0.0001		
**12mo S6K1-TSC1mKO**	<0.0001	<0.0001	<0.0001	0.003	<0.0001		

two-way ANOVA pS6-	6mo Control	28mo Control	6mo S6K1mKO	28mo S6K1mKO	12mo Control	12mo TSC1mKO	12mo S6K1-TSC1mKO
**6mo Control**		<0.0001		0.0046			0.0148
**28mo Control**	<0.0001		0.0005		0.0012		
**6mo S6K1mKO**				0.0005			
**28mo S6K1mKO**	0.0046						
**12mo Control**		0.0012					
**12mo TSC1mKO**							
**12mo S6K1-TSC1mKO**	0.0148						

Supplementary Table 2. List of PCR primers used in this study

	Forward	Reverse
Chrna1	TGGACCTATGACGGCTCTGT	GGAGTAGAACACCCAGTGCT
Chrnb1	ACGTTGCCCTGGACATCAAT	CAACCGAGAGGTTTGGGTCA
Chrne	CGCTATGAGGGAGGTTCCAC	GACAGTCTGGGCTAGCAGGA
Chrng	AGGCAGCGCAATGGATTAGT	TTACAGGCATCCACACAGGC
Musk	ACTGCGTGGAATGAGCTGAA	TCCATTGCCTGCCATTCCTT
Lrp4	GTGTGTGACGGGGACAATG	GCATTGCTCGTCACTGTTGT
NCAM	TGACGACTCCTCTACCCTCAC	AATCACAGCATCCTCCCCTTC
Runx1	GCCATGAAGAACCAGGTAGC	GCCGTCCACTGTGATTTTG
Gadd45a	GAAAGTCGCTACATGGATCAGT	AAACTTCAGTGCAATTTGGTTC
Noggin	TCTTGGCTACAGAGACCTGG	GAAGCCGGGTAACTTTTGACG
Myogenin	CTACAGGCCTTGCTCAGCTC	TGGGACCGAACTCCAGTG

Supplementary Table 3. List of RNAScope probes

**Gene**	**Species**	**Channel**	**Catalogue no.**
Chrne	Mouse	C3	832701
Eif4ebp1	Mouse	C1	439321

Supplementary Table 4
Statistical analysis of the number of AChR fragmentation (Fig. 4d).

**two-way ANOVA female**	**6mo Control**	**28mo Control**	**6mo 4EBP1mt-muscle**	**28mo 4EBP1mt-muscle**	**12mo Control**	**12mo TSC1mKO**	**12mo 4EBP1mt-TSC1mKO**
**6mo Control**		0.9997	0.1093	0.0086 **	>0.9999	<0.0001 ****	<0.0001 ****
**28mo Control**	0.9997		0.2227	0.0230 *	>0.9999	0.0001 ***	<0.0001 ****
**6mo 4EBP1mt-muscle**	0.1093	0.2227		0.9783	0.4399	0.2814	<0.0001 ****
**28mo 4EBP1mt-muscle**	0.0086 **	0.0230 *	0.9783		0.1009	0.7947	<0.0001 ****
**12mo Control**	>0.9999	>0.9999	0.4399	0.1009		0.0026 **	<0.0001 ****
**12mo TSC1mKO**	<0.0001 ****	0.0001 ***	0.2814	0.7947	0.0026 **		<0.0001 ****
**12mo 4EBP1mt-TSC1mKO**	<0.0001 ****	<0.0001 ****	<0.0001 ****	<0.0001 ****	<0.0001 ****	<0.0001 ****	

**two-way ANOVA Male**	6mo Control	28mo Control	6mo 4EBP1mt-muscle	28mo 4EBP1mt-muscle	12mo Control	12mo TSC1mKO	12mo 4EBP1mt-TSC1mKO
**6mo Control**		0.6134	0.0100 *	<0.0001 ****	>0.9999	0.0001 ***	<0.0001 ****
**28mo Control**	0.6134		0.2413	0.0099 **	0.9518	0.0069 **	<0.0001 ****
**6mo 4EBP1mt-muscle**	0.0100 *	0.2413		0.9704	0.0932	0.7867	0.0005 ***
**28mo 4EBP1mt-muscle**	<0.0001 ****	0.0099 **	0.9704		0.0052 **	0.9943	0.0047 **
**12mo Control**	>0.9999	0.9518	0.0932	0.0052 **		0.0031 **	<0.0001
**12mo TSC1mKO**	0.0001 ***	0.0069 **	0.7867	0.9943	0.0031 **		0.0967
**12mo 4EBP1mt-TSC1mKO**	<0.0001 ****	<0.0001 ****	0.0005 ***	0.0047 **	<0.0001	0.0967	

Supplementary Table 5

Statistical analysis of the number of post-synaptic myonuclei (Fig. 4e).

two-way ANOVA female	6mo Control	28mo Control	6mo 4EBP1mt-muscle	28mo 4EBP1mt-muscle	12mo Control	12mo TSC1mKO	12mo 4EBP1mt-TSC1mKO
**6mo Control**		0.5211	0.0175 *	0.0199 *	0.9933	>0.9999	0.0018 **
**28mo Control**	0.5211		0.0001 ***	0.0001 ***	0.9883	0.5556	<0.0001 ****
**6mo 4EBP1mt-muscle**	0.0175 *	0.0001 ***		>0.9999	0.0177 *	0.0812	0.9608
**28mo 4EBP1mt-muscle**	0.0199 *	0.0001 ***	>0.9999		0.0197 *	0.0893	0.9530
**12mo Control**	0.9933	0.9883	0.0177 *	0.0197 *		0.9847	0.0023 **
**12mo TSC1mKO**	>0.9999	0.5556	0.0812	0.0893	0.9847		0.0110 *
**12mo 4EBP1mt-TSC1mKO**	0.0018 **	<0.0001 ****	0.9608	0.9530	0.0023 **	0.0110 *	

**two-way ANOVA Male**	6mo Control	28mo Control	6mo 4EBP1mt-muscle	28mo 4EBP1mt-muscle	12mo Control	12mo TSC1mKO	12mo 4EBP1mt-TSC1mKO
**6mo Control**		0.5062	<0.0001 ****	0.0019 **	0.6007	0.9956	0.1568
**28mo Control**	0.5062		<0.0001 ****	<0.0001 ****	>0.9999	0.9949	0.0035 **
**6mo 4EBP1mt-muscle**	<0.0001 ****	<0.0001 ****		0.9041	<0.0001 ****	0.0005 ***	0.3173
**28mo 4EBP1mt-muscle**	0.0019 **	<0.0001 ****	0.9041		0.0001 ***	0.0085 **	0.9122
**12mo Control**	0.6007	>0.9999	<0.0001 ****	0.0001 ***		0.9843	0.0115 *
**12mo TSC1mKO**	0.9956	0.9949	0.0005 ***	0.0085 **	0.9843		0.1692
**12mo 4EBP1mt-TSC1mKO**	0.1568	0.0035 **	0.3173	0.9122	0.0115 *	0.1692	

Supplementary Table 6
Statistical analysis of Mander's colocalization coefficient (Fig. 4f).

**two-way ANOVA female**	**6mo Control**	**28mo Control**	**6mo 4EBP1mt-muscle**	**28mo 4EBP1mt-muscle**	**12mo Control**	**12mo TSC1mKO**	**12mo 4EBP1mt-TSC1mKO**
**6mo Control**		>0.9999	0.9997	0.8082	0.9875	0.8087	0.0319 *
**28mo Control**	>0.9999		0.9995	0.7869	0.9890	0.7865	0.0288 *
**6mo 4EBP1mt-muscle**	0.9997	0.9995		0.9691	0.9401	0.9690	0.1257
**28mo 4EBP1mt-muscle**	0.8082	0.7869	0.9691		0.4934	>0.9999	0.5382
**12mo Control**	0.9875	0.9890	0.9401	0.4934		0.4929	0.0162 **
**12mo TSC1mKO**	0.8087	0.7865	0.9690	>0.9999	0.4929		0.5386
**12mo 4EBP1mt-TSC1mKO**	0.0319 *	0.0288 *	0.1257	0.5382	0.0162 **	0.5386	

**two-way ANOVA Male**	6mo Control	28mo Control	6mo 4EBP1mt-muscle	28mo 4EBP1mt-muscle	12mo Control	12mo TSC1mKO	12mo 4EBP1mt-TSC1mKO
**6mo Control**		0.0754	0.0031 **	0.0081 **	0.7258	0.8797	0.0760
**28mo Control**	0.0754		0.5285	0.8247	0.9944	0.9897	0.9919
**6mo 4EBP1mt-muscle**	0.0031 **	0.5285		0.9985	0.3744	0.3915	0.9622
**28mo 4EBP1mt-muscle**	0.0081 **	0.8247	0.9985		0.6310	0.6347	0.9990
**12mo Control**	0.7258	0.9944	0.3744	0.6310		>0.9999	0.9140
**12mo TSC1mKO**	0.8797	0.9897	0.3915	0.6347	>0.9999		0.8996
**12mo 4EBP1mt-TSC1mKO**	0.0760	0.9919	0.9622	0.9990	0.9140	0.8996	

Supplementary Table 7

Statistical analysis of quantification of the percentages of NCAM-positive myofibers (Fig. 4g).

one-way ANOVA	3mo Control	28mo Control	3mo 4EBP1mt-muscle	28mo 4EBP1mt-muscle	12mo Control	12mo TSC1mKO	12mo 4EBP1mt-TSC1mKO
**3mo Control**		<0.0001 ####	0.0509	0.0004 ###	0.9807	<0.0001 ####	0.0160 #
**28mo Control**	<0.0001 ####		0.0572	>0.9999	0.0006 ###	0.7120	0.4065
**3mo 4EBP1mt-muscle**	0.0509	0.0572		0.1877	0.3303	0.0081 ##	0.9820
**28mo 4EBP1mt-muscle**	0.0004 ###	>0.9999	0.1877		0.0039 ##	0.7549	0.6470
**12mo Control**	0.9807	0.0006 ###	0.3303	0.0039 ##		0.0002 ###	0.1209
**12mo TSC1mKO**	<0.0001 ####	0.7120	0.0081 ##	0.7549	0.0002 ###		0.0669
**12mo 4EBP1mt-TSC1mKO**	0.0160 #	0.4065	0.9820	0.6470	0.1209	0.0669	
