# Supplementary material for: Muscle 4EBP1 activation modifies the structure and function of the neuromuscular junction in mice
Source: Nat Commun. 2022 Dec 17;13:7792. doi: 10.1038/s41467-022-35547-0 (PMC9758177; doi:10.1038/s41467-022-35547-0)
Supplement: Supplementary file 3 — Reporting Summary [file 41467_2022_35547_MOESM3_ESM.pdf]

nature portfolio

Corresponding author(s): Shih-Yin, Tsai

Last updated by author(s): Aug 31, 2022

Reporting Summary

Nature Portfolio wishes to improve the reproducibility of the work that we publish. This form provides structure for consistency and transparency in reporting. For further information on Nature Portfolio policies, see our Editorial Policies and the Editorial Policy Checklist.

Statistics

For all statistical analyses, confirm that the following items are present in the figure legend, table legend, main text, or Methods section.

n/a	Confirmed	
☐	☒	The exact sample size (*n*) for each experimental group/condition, given as a discrete number and unit of measurement
☐	☒	A statement on whether measurements were taken from distinct samples or whether the same sample was measured repeatedly
☐	☒	The statistical test(s) used AND whether they are one- or two-sided<br>*Only common tests should be described solely by name; describe more complex techniques in the Methods section.*
☒	☐	A description of all covariates tested
☐	☒	A description of any assumptions or corrections, such as tests of normality and adjustment for multiple comparisons
☐	☒	A full description of the statistical parameters including central tendency (e.g. means) or other basic estimates (e.g. regression coefficient) AND variation (e.g. standard deviation) or associated estimates of uncertainty (e.g. confidence intervals)
☐	☒	For null hypothesis testing, the test statistic (e.g. *F*, *t*, *r*) with confidence intervals, effect sizes, degrees of freedom and *P* value noted<br>*Give P values as exact values whenever suitable.*
☒	☐	For Bayesian analysis, information on the choice of priors and Markov chain Monte Carlo settings
☒	☐	For hierarchical and complex designs, identification of the appropriate level for tests and full reporting of outcomes
☒	☐	Estimates of effect sizes (e.g. Cohen's *d*, Pearson's *r*), indicating how they were calculated

Our web collection on statistics for biologists contains articles on many of the points above.

Software and code

Policy information about availability of computer code

Data collection	Microscopy: Olympus confocal microscope FV100 or FV3000 and TissueFAXS Slide Scanner (TissueGnostics) and TissueFAXSViewer software.<br>Western Blotting: Thermofisher iBright Imaging Systems FL1500<br>RT-PCR: Roch Light Cycler 480 instrument II
Data analysis	Image analysis was performed using Imaris Software. The myofiber size of MyHC isoform populations was calculated using image J.<br>Statistical analyses were performed using GraphPad Prism 9 software.

For manuscripts utilizing custom algorithms or software that are central to the research but not yet described in published literature, software must be made available to editors and reviewers. We strongly encourage code deposition in a community repository (e.g. GitHub). See the Nature Portfolio guidelines for submitting code & software for further information.

Data

Policy information about availability of data

All manuscripts must include a data availability statement. This statement should provide the following information, where applicable:

- Accession codes, unique identifiers, or web links for publicly available datasets
- A description of any restrictions on data availability
- For clinical datasets or third party data, please ensure that the statement adheres to our policy

The data availability statement is given in the manuscript.

Human research participants

Policy information about studies involving human research participants and Sex and Gender in Research.

Reporting on sex and gender	NA
Population characteristics	NA
Recruitment	NA
Ethics oversight	NA

Note that full information on the approval of the study protocol must also be provided in the manuscript.

Field-specific reporting

Please select the one below that is the best fit for your research. If you are not sure, read the appropriate sections before making your selection.

[x] Life sciences [ ] Behavioural & social sciences [ ] Ecological, evolutionary & environmental sciences

For a reference copy of the document with all sections, see nature.com/documents/nr-reporting-summary-flat.pdf

Life sciences study design

All studies must disclose on these points even when the disclosure is negative.

Sample size	Sample sizes were chosen based on previous experiences (doi: 10.1016/j.celrep.2016.07.029. ; doi: 10.1172/JCI77361.).
Data exclusions	Data points were not excluded.
Replication	All experiments were replicated on numerous animals at different times. The quantification of NMJ were reproducible by 2-3 independent researchers.
Randomization	Litter-mate control were used for all experiments. Multiple litters were generated over t
Blinding	All histology analysis were quantified blind.

Reporting for specific materials, systems and methods

We require information from authors about some types of materials, experimental systems and methods used in many studies. Here, indicate whether each material, system or method listed is relevant to your study. If you are not sure if a list item applies to your research, read the appropriate section before selecting a response.

Materials & experimental systems

n/a	Involved in the study	
[ ]	[x]	Antibodies
[x]	[ ]	Eukaryotic cell lines
[x]	[ ]	Palaeontology and archaeology
[ ]	[x]	Animals and other organisms
[x]	[ ]	Clinical data
[x]	[ ]	Dual use research of concern

Methods

n/a	Involved in the study	
[x]	[ ]	ChIP-seq
[x]	[ ]	Flow cytometry
[x]	[ ]	MRI-based neuroimaging

Antibodies

Antibodies used	Primary Antibody:<br>rabbit anti-neurofilament (1:1500; Cell Signaling, 2837),<br>rabbit anti-synaptophysin (1:300, Invitrogen PA1-1043),<br>rabbit anti-Phospho-S6S240+244 (1:100, Cell Signaling, 2215),<br>rabbit-anti NCAM (1:100, Millipore Merck, AB5032),<br>rat anti-laminin (1:300, Abcam, ab11576),<br>mouse IgG1-anti-Pax7 (1:50; Developmental Studies Hybridoma Bank),

mouse IgG1-anti-MYH2 (1:60, Developmental Studies Hybridoma Bank),
mouse IgG2b-anti-MYH7 (1:25, Developmental Studies Hybridoma Bank),
mouse IgM-anti-and MYH4 (1:75, Developmental Studies Hybridoma Bank),
(goat-anti-mouse IgG1-AF488 [1:400, Invitrogen, A-21121], goat-anti-mouse IgG2b-AF350 [1:200, Invitrogen, A-21140] and goat anti-mouse IgM-AF594 [1:400, Invitrogen, A-21044],
α- p70 S6 kinase 1 (Cell Signaling, 9202),
α- p70 S6 kinase 2 (Cell Signaling, 14130),
α-S6 Ribosomal Protein (Cell Signaling, 2217),
α-phospho-S6 ribosomal protein Ser240+244 (Cell Signaling, 2215),
α- 4EBP1 (Cell Signaling, 9452),
α phospho-4EBP1 Thr37+46 (Cell Signaling, 2855),
α- Akt (Cell Signaling, 4691),
α phospho-AKT Ser473(Cell Signaling, 4060),
α-actinin (Cell Signaling, 3134),
α phospho-p70 S6 kinase 2 Ser423 (sigma, SAB4301595),
α TSC1 (Axil Scientific, A300-316A)
Secondary Antibody:
anti-rabbit IgG Alexa Fluor (AF) 564 (1:1000; Invitrogen, A10042),
anti-rabbit IgG AF-594 (1:500, Invitrogen, A11012),
anti-rat IgG AF-647 (1:500, Invitrogen, A21247),
goat-anti-mouse IgG1-AF488 [1:400, Invitrogen, A-21121],
goat-anti-mouse IgG2b-AF350 [1:200, Invitrogen, A-21140],
goat anti-mouse IgM-AF594 [1:400, Invitrogen, A-21044]),
sheep anti-mouse IgG-HRP (Amersham, NA-931),
donkey anti-Rabbit IgG-HRP (Amersham, NA-934).

Validation

The antibodies used in the study were validated by the companies.

Animals and other research organisms

Policy information about studies involving animals; ARRIVE guidelines recommended for reporting animal research, and Sex and Gender in Research

Laboratory animals	Strain: C57BL/6J.<br>TSC1mKO (TSC1f/f; Ckmm-Cre), S6K1mKO (S6K1f/f; Ckmm-Cre), S6K1-TSC1mKO (TSC1f/f; S6K1f/f; Ckmm-Cre), 4EBP1mt-muscle (4EBP1mt; Ckmm-Cre) and 4EBP1mt-TSC1mKO (TSC1f/f; 4EBP1mt; Ckmm-Cre).
Wild animals	NA
Reporting on sex	Both males and females are included and specified in the study.
Field-collected samples	NA
Ethics oversight	All experimental procedures involving the use of animals were reviewed and approved by the IACUC of the National University of Singapore.

Note that full information on the approval of the study protocol must also be provided in the manuscript.
